# "In sickness and in health": sickness absenteeism in Federal Highway Patrol Officers in the state of Rio Grande do Sul, Brazil

**DOI:** 10.47626/1679-4435-2023-1068

**Published:** 2024-02-16

**Authors:** José Rossy e Vasconcelos Júnior, Eduardo Frio Marins, Eduardo Lucia Caputo

**Affiliations:** 1 Polícia Rodoviária Federal, Brasília, DF, Brazil; 2 Programa de Pós-Graduação em Educação Física, Universidade Federal de Pelotas, Pelotas, RS, Brazil

**Keywords:** police, mental health, sick leave, polícia, saúde mental, licença médica

## Abstract

**Introduction:**

Police activity exposes the workers to several conditions that can cause physical and mental health problems, leading to sickness absenteeism.

**Objectives:**

To describe the reasons for sickness absenteeism in Federal Highway Patrol Officers in the state of Rio Grande do Sul, Brazil.

**Methods:**

We used secondary data from official records of sickness absenteeism of Federal Highway Patrol Officers in Rio Grande do Sul. Sickness absenteeism was classified according to the International Classification of Diseases - 10th Revision. The variables analyzed were: reasons for sickness absenteeism, by code and category of the International Classification of Diseases - 10th Revision, and days absent from work by International Classification of Diseases - 10th Revision code. Descriptive data were reported using frequency distribution and measures of central tendency and dispersion. We used the Kruskal-Wallis test to compare the days absent from work between the International Classification of Diseases - 10th Revision codes.

**Results:**

The most common reason for sickness absenteeism was diseases of the musculoskeletal system and connective tissue (24.6%). Mental and behavioral disorders were associated with the highest number of days absent from work (32.6 ± 19.9 days). Within the most prevalent disease groups, depressive disorders (30%), fractures (30%), and low back pain (15.9%) were the disease categories with the highest frequencies.

**Conclusions:**

Sickness absenteeism among Federal Highway Patrol Officers is predominantly related to diseases of the musculoskeletal system and connective tissue, and prolonged sick leave is due mainly to mental and behavioral disorders. Therefore, this police organization needs to promote and implement prevention programs to manage the main morbidities.

## Abstract (pt)

**Introdução:**

A atividade policial expõe seus trabalhadores a diversas condições que podem ocasionar problemas de saúde tanto físicos quanto mentais e consequente absenteísmo do profissional.

**Objetivos:**

Descrever os motivos de absenteísmo-doença em agentes da Polícia Rodoviária Federal do Rio Grande do Sul.

**Métodos:**

Utilizamos dados secundários de registros oficiais de absenteísmo do trabalho de agentes da Polícia Rodoviária Federal do Rio Grande do Sul. Os absenteísmos-doença foram classificados conforme a Classificação Internacional de Doenças - 10ª Revisão. As variáveis analisadas foram: motivos de absenteísmo-doença, por grupo e categoria da Classificação Internacional de Doenças - 10ª Revisão e dias de afastamento por grupo da Classificação Internacional de Doenças - 10ª Revisão. Dados descritivos foram reportados através de distribuição de frequências e de tendência central e dispersão. Utilizamos o teste de Kruskall-Wallis para comparação dos dias de afastamento entre os grupos de Classificação Internacional de Doenças - 10ª Revisão.

**Resultados:**

A maior prevalência de afastamentos foi por doenças do sistema osteomuscular e do tecido conjuntivo (24,6%). O grupo de doenças dos transtornos mentais e de comportamento foi o que apresentou maior número de dias de afastamento (32,6 ± 19,9 dias). Entre os grupos mais prevalentes, transtornos depressivos (30%), fraturas (30%) e dor lombar (15,9%) foram as categorias de doença que apresentaram maiores frequências.

**Conclusões:**

O absenteísmo-doença entre os agentes da Polícia Rodoviária Federal predomina por doenças do sistema osteomuscular e do tecido conjuntivo, bem como possui elevada duração por transtornos mentais e de comportamento. Assim, existe uma necessidade dessa organização policial de promover a prevenção e implementação de programas de gerenciamento dessas principais morbidades.

## INTRODUCTION

Sickness absenteeism is defined as non-attendance at work due to incapacity other than that resulting from pregnancy or imprisonment,^1^ and is considered a complex phenomenon,^2^ having different causes (eg_;_ musculoskeletal or psychosocial complaints). The sickness absenteeism occurrence and duration can be influenced by a number of factors, such as demographic, behavioral, mental health, personal, organizational, and work-related factors.^3^ Police work, such as that performed by Federal Highway Patrol Officers (FHPOs), exposes the workers to mentally stressful situations (eg, armed clashes, violence, and threats to life) and physically demanding tasks (eg, carrying heavy protective gear), which are considered predictors of sickness absenteeism.^3^

Data from a retrospective cross-sectional study of military police officers (MPOs) from Recife (state of Pernambuco, Brazil) indicated that diseases of the musculoskeletal system and connective tissue had a higher prevalence of sickness absenteeism (20%) and were the main cause of prolonged leave.^4^ Conversely, a study of MPOs from Marília (state of São Paulo, Brazil) revealed trauma while on duty as the main cause of prolonged leave.^5^ This scenario indicates a lack of studies investigating the main reasons for sickness absenteeism in police populations,^6,7^ especially in non-military police, such as FHPOs.

The consequences of sickness absenteeism involve increased expenses and reduced productivity^8^ negatively impacting the state and society. Identifying the main reasons for sickness absenteeism among police officers is important to provide information on their health conditions, helping guide occupational health policies in police agencies. Therefore, the primary objective of this study was to describe the prevalence of the main causes of sickness absenteeism in FHPOs in the state of Rio Grande do Sul, Brazil. The second objective was to investigate the difference in the number of days absent from work due to treatment between disease groups.

## METHODS

We conducted a cross-sectional observational epidemiological study with retrospective data collection and a descriptive-analytical approach.^9^

The target population of this study consisted of all FHPOs in the state of Rio Grande do Sul, Brazil, who were granted sick leave to treat a health condition (medical certificates) in 2017. A database containing records of all sick leaves to treat a health condition (medical certificates) was provided by the People Management Sector of the Federal Highway Patrol Superintendency in the state of Rio Grande do Sul *(Seção de Gestão de Pessoas/Polícia Rodoviária Federal - Rio Grande do Sul,* SGP/PRF-RS). This superintendency encompasses 13 police stations in different cities in Rio Grande do Sul, where the FHPOs perform their duties on federal highways as well as administrative and management activities.

This study included all justified reasons for sick leave (sickness absenteeism) that occurred from January 1 to December 31, 2017. Justified reasons were any reason for sick leave supported by medical certification of illness from any FHPO assigned to any of the 13 police stations of the Federal Highway Patrol Superintendency in the state of Rio Grande do Sul. The following medical certificates were excluded from the database:

**a)** sick leave to care for a family member;
**b)** non-police employees (administrative career); or
**c)** certificates lacking the International Classification of Diseases - 10th Revision (lCD-10) codes (categories).

The following variables were analyzed in this study: sex (male/female), date of birth, education (high school/higher education), date of joining the police, ICD-10 group and category (code), and total number of days absent from work as reported in the medical certificate. The FHPO s age and length of service were obtained by subtracting the year in which the study was conducted (2017) by the year of birth and the year of joining the police, respectively.

Anonymity was preserved by using only codes to refer to the FHPOs in the database (making identification impossible). Based on the archival nature of this study (retrospective and anonymized data) and the resolutions of the Brazilian National Health Council (Conselho Nacional de Saúde, CNS),^10^ the study was exempt from research ethics committee approval.

The data were transferred to Stata and subsequently subjected to descriptive statistical analysis. Numerical data are presented as measures of central tendency (mean, SD, median, and IQR), and categorical data as absolute and relative frequencies. Given the asymmetric distribution of the variable 'days absent from work/ the Kruskal-Wallis test was used to assess differences in this variable between the ICD-10 codes. All analyses were performed using Stata 15, and a p-value < 0.05 was considered statistically significant.

## RESULTS

**Figure 1.**
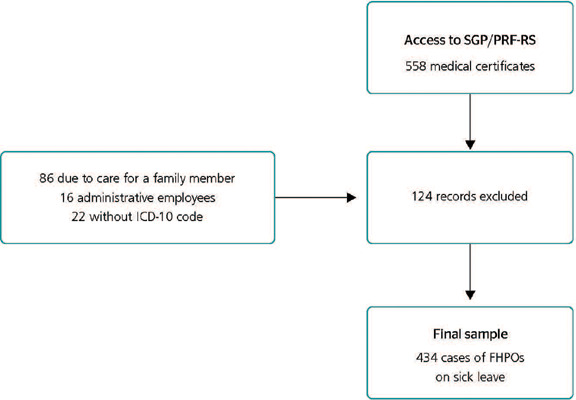
Participant flowchart. FHPO = Federal Highway Patrol Officer; ICD-10: International Classification of Diseases - 10th Revision; SGP/PRF-RS = People Management Sector of the Federal Highway Patrol Superintendency in the state of Rio Grande do Sul.

A total of 558 sick leave records were provided by the People Management Sector in 2017. After applying the eligibility criteria, 434 records were included. Figure 1 provides the participant eligibility flowchart.

A total of 217 FHPOs were absent from work due to illness (sickness absenteeism) at some point in 2017. Mean participant age was 42.5 ±7.1 years, most were men (89.4%) and had completed higher education (62.7%). The mean length of service as an FHPO was 13.2 ± 7.9 years, and the median number of days absent from work was 7 (IQR, 3-30) days.

**Figure 2.**
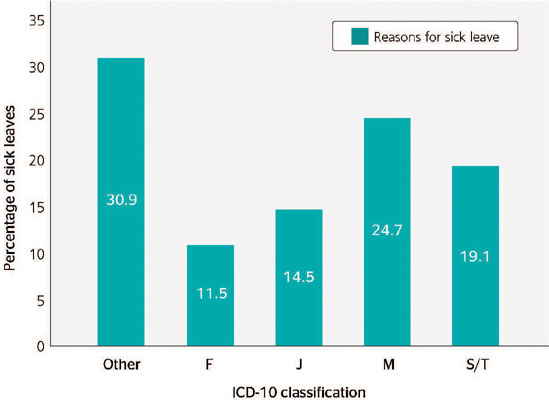
Relative frequency (%) of reasons for sickness absenteeism of Federal Highway Patrol Officers (FHPOs) in the state of Rio Grande do Sul in 2017, according to the most prevalent International Classification of Diseases - 10th Revision (ICD-10) codes. F = Mental, behavioral and neurodevelopmental disorders; J = Diseases of the respiratory system; M = Diseases of the musculoskeletal system and connective tissue; S/T = Injury, poisoning and certain other consequences of external causes; Other = other ICD-10 diseases.

Figure 2 shows the relative frequency data of medical certificates grouped according to the ICD-10 codes. The most prevalent sick leave groups were ICD-10 M (Diseases of the musculoskeletal system and connective tissue, 24.7%), S/T (injury, poisoning and certain other consequences of external causes, 19.1%), J (Diseases of the respiratory system, 14.5%), and F (Mental, behavioral and neurodevelopmental disorders, 11.5%). Complete data on reasons for sick leave grouped by ICD-10 code can be found in Annex 1.

**Figure 3.**
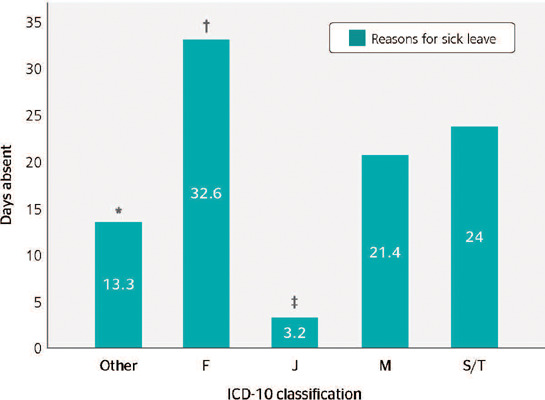
Mean days of sickness absenteeism of Federal Highway Patrol Officers (FHPOs) in the state of Rio Grande do Sul in 2017, according to the most prevalent International Classification of Diseases - 10th Revision (ICD-10) codes. F = Mental, behavioral and neurodevelopmental disorders; J = Diseases of the respiratory system; M = Diseases of the musculoskeletal system and connective tissue; S/T = Injury, poisoning and certain other consequences of external causes; Other = other ICD-10 diseases. * Significantly (p < 0.05) different from groups F, J, M, and S/T;† Significantly (p < 0.05) different from groups J and M; ‡ Significantly (p < 0.05) different from groups M and S/T.

The mean number of days absent from work according to the ICD-10 codes are shown in Figure 3. Overall, the groups "Diseases of the musculoskeletal system and connective tissue" (ICD-M), "Mental, behavioral and neurodevelopmental disorders" (ICD-F), and "Injury, poisoning and certain other consequences of external causes" (ICD-S/T) had longer sick leave than the "Diseases of the respiratory system" group (ICD-J) and all other groups combined. The "Mental, behavioral and neurodevelopmental disorders" group (ICD-F) was the reason with the highest number of days absent (32.6 ± 19.9 days), showing significant difference compared (p < 0.05) to groups "Diseases of the musculoskeletal system and connective tissue" (ICD-M) and "Diseases of the respiratory system" (ICD-J).

Table 1 shows the most prevalent categories by ICD-10 code. The most prevalent causes of sick leave within each ICD-10 code included depressive disorders (30%), fractures (30%), and low back pain (15.9%).

**Table 1.** Absolute and relative frequency (%) for sickness absenteeism of Federal Highway Patrol Officers (FHPOs) in the state of Rio Grande do Sul in 2017, according to the main categories of diseases by International Classification of Diseases - 10th Revision (ICD-10) code

Categories	n(%)
ICD-F - Mental, behavioral and neurodevelopmental disorders (n = 50)	
Recurrent depressive disorder	15 (30.0)
Major depressive episode	8 (16.0)
Acute stress reaction	6 (12.0)
Other	21 (42.0)
ICD-M - Diseases of the musculoskeletal system and connective tissue (n = 107)	
Low back pain	17 (15.9)
Rotator cuff syndrome	15 (14.0)
Other	75 (70.1)
ICD-S/T - Injury poisoning and certain other consequences of external causes (n = 80)	
Fractures	24 (30.0)
Trauma	8 (10.0)
Other	48 (60.0)

## DISCUSSION

Musculoskeletal problems were the main cause of sickness absenteeism among FHPOs. However, although this was the most frequent reason for sick leave, mental disorders were responsible for longer sick leave in this population. Depressive disorders, fractures, and low back pain were among the main reasons for sickness absenteeism in FHPOs.

The main cause of sick leave (diseases of the musculoskeletal system and connective tissue - ICD-M) reported in this study (24.6%) was similar to that found in MPOs from Recife (state of Pernambuco, Brazil).^4^ Between 2009 and 2013, 20% of reported sick leaves were due to disorders of the musculoskeletal system.^4^ This predominance can be explained by the occupational conditions inherent in the police work, such as the use of heavy personal protective equipment attached to the body (eg, bulletproof vests and weapons) and continuous inadequate postures (eg, standing or sitting) for long periods. However, longitudinal studies are needed to confirm these hypotheses.

Furthermore, low back pain was the third most prevalent disease (15.9%), consistent with the study by Martins et al.,^11^ conducted in MPOs in the metropolitan area of Belém (state of Pará, Brazil). Low back pain is considered a global public health problem,^12^ affecting police officers, even chronically.^13^ Data from a recent study identified a high prevalence (67.8%) of chronic low back pain in FHPOs from the state of Rio Grande do Sul.^13^ Despite supporting the sick leave data found, it should be noted that many police officers may be working with low back pain,^13^ which limits performance and productivity while on duty, being a type of absenteeism in which employees show up for work but are unable to fully perform their tasks (presenteeism).^14^ However, there are few studies demonstrating a relationship between sickness absenteeism and low back pain in police populations, such as that indicated by a systematic review.^6^

The difference found in the higher number of days absent between the group of mental, behavioral and neurodevelopmental disorders (ICD-F) and the groups of diseases of the respiratory system (ICD-J) and diseases of the musculoskeletal system and connective tissue (ICD -M) can be explained by occupational situations with a high level of emotional stress and traumatic potential faced by FHPOs. Examples include the following: incidents resulting in death; suffering or witnessing verbal, physical, and/or psychological harm; legal insecurity and derogatory social judgments; and feeling pressured by institutional managerial demands.^15,16^

As a consequence, police officers can develop mental disorders (eg, depressive episodes, stress, anxiety, and post-traumatic stress disorder), as confirmed by the most prevalent disease category in this study (depressive disorder). These disorders, due to difficulties in identifying their signs and symptoms as well as the resistance to seeking treatment and the stigma socially associated with these diseases, are diagnosed and treated late, often at a chronic stage of disease, leading to longer duration of treatment and recovery of health status.^17^

However, data involving other police officers, such as MPOs, do not corroborate the findings of the present study. Quirino et al.,^4^ analyzing a sick leave database of MPOs in Recife (state of Pernambuco, Brazil), identified ICD-M and ICD-S as the disease groups leading to the highest number of days absent: 12,979 and 10,387 days, respectively. Such differences may be explained by the distinct characteristics between the police categories, such as the place of operation (FHPOs on highways and MPOs in neighborhoods, favelas, and games and events) and the profile of the police officers, since the percentage of MPOs who work a second job on their days off (mainly in private security companies) is very high.^5,18^

The limitations of our study need to be addressed. First, the 1-year period as well as the analysis of FHPOs only from Rio Grande do Sul prevent us to extrapolate the results. Second, it is possible that FHPOs continue attending work while ill (ie, presenteeism), and a possible selection bias cannot be ruled out. Further studies should cover other time points as well as FHPOs from other states, since the FHP is a police institution present in all Brazilian states and the Federal District.

We believed that the results of this study will positively impact police categories, increasing the interest of police institutions' decision-makers in the topic. Furthermore, they can assist planning and implementing evidence-based occupational health policies to reduce sick leaves. For police officers, acknowledging the main reasons for sickness absenteeism and their proportions may serve as a warning to the need for preventive care in relation to police work, encouraging healthier lifestyle habits.

Finally, identifying a high prevalence of sickness absenteeism related to diseases of the musculoskeletal system and connective tissue as well as a high number of days absent from work due to mental and behavioral disorders in FHPOs may encourage researchers and occupational health sectors to develop programs aimed at disease prevention, health promotion, treatment, and health surveillance in order to reduce the incidence and frequency of these morbidities in this understudied population in Brazil. Furthermore, it may guide institutional strategies and public policies for the prevention of these diseases and the promotion of comprehensive health among the workforce, reducing both presenteeism and sickness absenteeism and contributing to providing society with higher quality public security services.

## CONCLUSIONS

FHPOs in southern Brazil have sickness absenteeism predominantly related to diseases of the musculoskeletal system and connective tissue as well as prolonged sick leave due to mental and behavioral disorders. Furthermore, sick leaves are caused mainly by depressive disorders, fractures, and low back pain. Therefore, police organizations need to promote and implement prevention programs to manage the main morbidities.
